# Less is more: improving cell-type identification with augmentation-free single-cell RNA-Seq contrastive learning

**DOI:** 10.1093/bioinformatics/btaf437

**Published:** 2025-08-05

**Authors:** Ibrahim Alsaggaf, Daniel Buchan, Cen Wan

**Affiliations:** School of Computing and Mathematical Sciences, Birkbeck, University of London, London WC1E 7HX, United Kingdom; Department of Computer Science, University College London, London WC1E 6BT, United Kingdom; School of Computing and Mathematical Sciences, Birkbeck, University of London, London WC1E 7HX, United Kingdom

## Abstract

**Motivation:**

Cell-type identification is one of the most important tasks in single-cell RNA Sequencing (scRNA-Seq) analysis. Recent research has revealed contrastive learning’s great potential in handling multiple cell-type identification tasks.

**Results:**

In this work, we proposed a novel augmentation-free scRNA-Seq contrastive learning (AF-RCL) algorithm, which simplifies the conventional data augmentation operation and adopts a new contrastive learning loss function. A large-scale empirical evaluation suggests that AF-RCL not only outperformed other contrastive learning-based cell-type identification methods but also obtained state-of-the-art predictive performance compared with other well-known cell-type identification methods. Further analysis also shows AF-RCL’s advantages in learning high-quality discriminative feature representations based on scRNA-Seq expression profiles.

**Availability and implementation:**

The source code is available at https://doi.org/10.6084/m9.figshare.28830311.v1 and at https://github.com/ibrahimsaggaf/AFRCL. The pre-trained AF-RCL encoders can be downloaded from https://doi.org/10.5281/zenodo.15109736, and the scRNA-Seq datasets used in this work can be downloaded from https://doi.org/10.5281/zenodo.8087611.

## 1 Introduction

Single-cell RNA Sequencing (scRNA-Seq) has greatly boosted the understanding and discovery of complex biological systems at a single-cell level, such as gene-regulatory logic (Iacono *et al.* 2019, Zhang *et al.* 2023, Yuan and Duren 2024), cell–cell communication (Tanay and Regev 2017, Almet *et al.* 2021, Wilk *et al.* 2024), and genomics function (Croucher *et al.* 2021, Salehi *et al.* 2021, Dufva *et al.* 2023). As one of the fundamental prerequisites of scRNA-Seq analysis, accurate cell-type identification plays a crucial role on elucidating the heterogeneity of cell composition in tissue samples. Many machine learning-based computational methods have been proposed to automatically annotate cell-type labels (Abdelaal *et al.* 2019), such as scPred (Alquicira-Hernandez *et al.* 2019), ACTINN (Ma and Pellegrini, 2020), and SingleCellNet (Tan and Cahan 2019), which show encouraging predictive performance, despite the well-known challenges of high-dimensionality and high-sparsity of scRNA-seq expression profiles. More recently, Alsaggaf *et al.* (2024) proposed a new contrastive learning-based cell-type identification method that successfully obtained the state-of-the-art predictive accuracy and demonstrated a great potential on the further improvement of cell-type identification methods.

Contrastive learning is an emerging representation learning paradigm that aims to learn a type of discriminative distribution, where all samples are projected uniformly in a target hypersphere. The conventional self-supervised contrastive learning methods like SimCLR (Chen *et al.* 2020b) exploit data augmentation strategies to create different data sample views, which are pulled closer if they are similar but pushed apart if they are dissimilar. The more recently proposed supervised contrastive learning paradigm (Khosla *et al.* 2020) also creates data sample views, but its learning process exploits pre-defined class label information—those views bearing the same class labels are pulled closer whilst the views are pushed apart if they bear different class labels. Contrastive learning has already achieved success in many tasks like image processing (He *et al.* 2020, Chen *et al.* 2020b) and natural language processing (Qu *et al.* 2020, Zhang *et al.* 2022). It has also been used to deal with multiple scRNA-Seq analysis tasks, e.g. scRNA-Seq clustering analysis (Ciortan and Defrance 2021, Wan *et al.* 2022), batch effects removal (Han *et al.* 2022, Wang *et al.* 2022), and data integration (Xu *et al.* 2022, Yang *et al.* 2022). In addition, Alsaggaf *et al.* (2024) proposed a novel Gaussian noise augmentation-based contrastive learning method to identify different cell-types using scRNA-Seq expression profiles.

Data augmentation plays a crucial role in contrastive learning, which relies on augmented sample sets to derive learning gradients. Depending on data types, different augmentation strategies were adopted for contrastive learning purposes. For example, in terms of image data, the most common approaches are image rotating, Gaussian blurring, and image cropping (Chen *et al.* 2020b,c, Khosla *et al.* 2020, Kang *et al.* 2021). When dealing with natural language data, paraphrasing and word replacement are usually used to create more diverse views (Qu *et al.* 2020). In terms of graph data, the conventional augmentation methods usually adopt stochastic approaches to perturb or drop vertices to create similar but different graphs as views (You *et al.* 2020, Wang *et al.* 2022). In terms of scRNA-Seq data, augmentation approaches that are based on random genes masking and Gaussian noise addition were proposed recently. For example, Ciortan and Defrance (2021) and Wan *et al.* (2022) proposed to generate different views of scRNA-Seq data by masking an arbitrary set of genes. Alsaggaf *et al.* (2024) and Xu *et al.* (2022) proposed adding random Gaussian noise vectors to cells’ scRNA-Seq expression profiles to create views.

The aforementioned conventional contrastive learning methods all rely on augmented sample sets to generate learning gradients so that the encoder network can learn discriminative representations. Some recent research has revealed an alternative contrastive learning paradigm, i.e. augmentation-free contrastive learning, which does not rely on any augmented sample sets. However, the augmentation-free contrastive learning paradigm mainly demonstrated its superb performance when handling graph-based data, due to the intrinsic structural information. For example, the most common approach is to exploit pre-defined node connectivity information and node similarity measurement to select positive sample (Lee *et al.* 2022, Li *et al.* 2024, Zhao *et al.* 2024). In this work, we proposed a novel augmentation-free scRNA-Seq contrastive learning (AF-RCL) algorithm which outperformed all recently proposed augmentation-based scRNA-Seq contrastive learning methods and successfully obtained higher predictive performance than other state-of-the-art cell-type identification methods.

## 2 Proposed methods

### 2.1 Augmentation-free single-cell RNA-Seq contrastive learning

**Figure 1.**
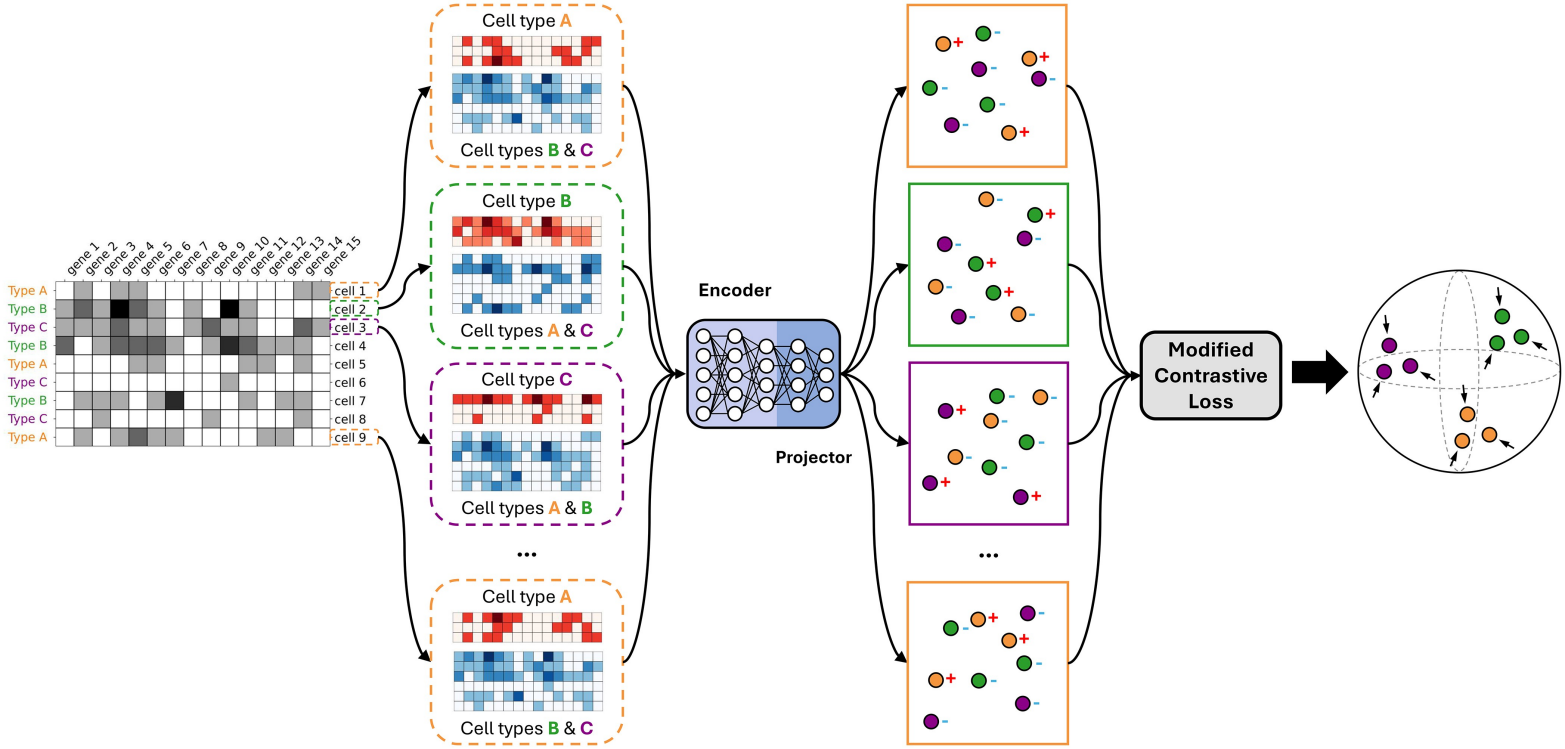
The flow chart for the augmentation-free single-cell RNA-Seq contrastive learning (AF-RCL) framework.

In general, as shown in Fig. 1, the proposed AF-RCL framework learns a type of discriminative feature representations, where different cell-types are distinct from each other. Given a set of cells described by scRNA-Seq expression profiles (i.e. the grey matrix), for each cell (instance), AF-RCL creates one pair of positive and negative cell sets, as denoted by red and blue matrices, respectively. The positive cell set consists of those cells belonging to the same cell-type as the target cell, whilst all other cells belonging to different cell-types to the target cell are included in the negative cell set. Those different pairs of positive and negative cell sets are then used as inputs for two neural networks (i.e. an encoder and a projector) to learn the discriminative feature representations using a modified contrastive learning loss function, without any data augmentation operation. To deal with the cell-type identification tasks, we used the AF-RCL-learned feature representations to train multi-class support vector machines.

The pseudocode of the proposed AF-RCL method is shown in Algorithm 1, where AF-RCL eventually outputs a trained encoder $E^{*}$ by taking five inputs, i.e. a set of batches B for a given training cell set X, a label set Y denoting different cell-types, a pair of untrained encoder E and projector P, and a pre-defined temperature hyperparameter τ. From lines 1 to 23, AF-RCL optimises the encoder and the projector after processing each batch $B_{k}$. In line 2, AF-RCL first creates an empty variable to store the loss function value $L_{B}$ for the batch $B_{k}$. Then it processes each cell in turns from lines 3–20. For each cell $x_{i}$, AF-RCL creates three variables, i.e. an empty variable $L_{i}$ to store the loss function value for $x_{i}$, two empty sets $H_{i}^{+}$ and $H_{i}^{-}$ for storing the projections of positive and negative cells for $x_{i}$, respectively. From lines 7 to 16, AF-RCL iterates all cells in $B_{k}$ to select the positive and negative cells for $x_{i}$ by considering those cells’ pre-defined cell type labels. For those cells bearing the same cell-type label as $x_{i}$, AF-RCL considers them as positive cells and adds their projections to $H_{i}^{+}$. *Vice versa*, AF-RCL considers those cells as negative if they bear different cell-type labels to $x_{i}$. The projections of those negative cells are added to $H_{i}^{-}$. After obtaining the complete projection sets for both positive and negative cells, AF-RCL also generates a projection for the target cell $x_{i}$, which is then used as one of the inputs to compute the loss function value $L_{i}$ (line 18). In line 19, AF-RCL increments the batch loss function value $L_{B}$, and the accumulated loss function value $L_{B}$ for the entire batch $B_{k}$ is used to optimise the encoder and the projector (lines 21–22). Note that, as AF-RCL does not use any augmented cell instances, the value of $L_{B}$ will be normalized by *m—*the number of cells in $B_{k}$.

**Figure:**
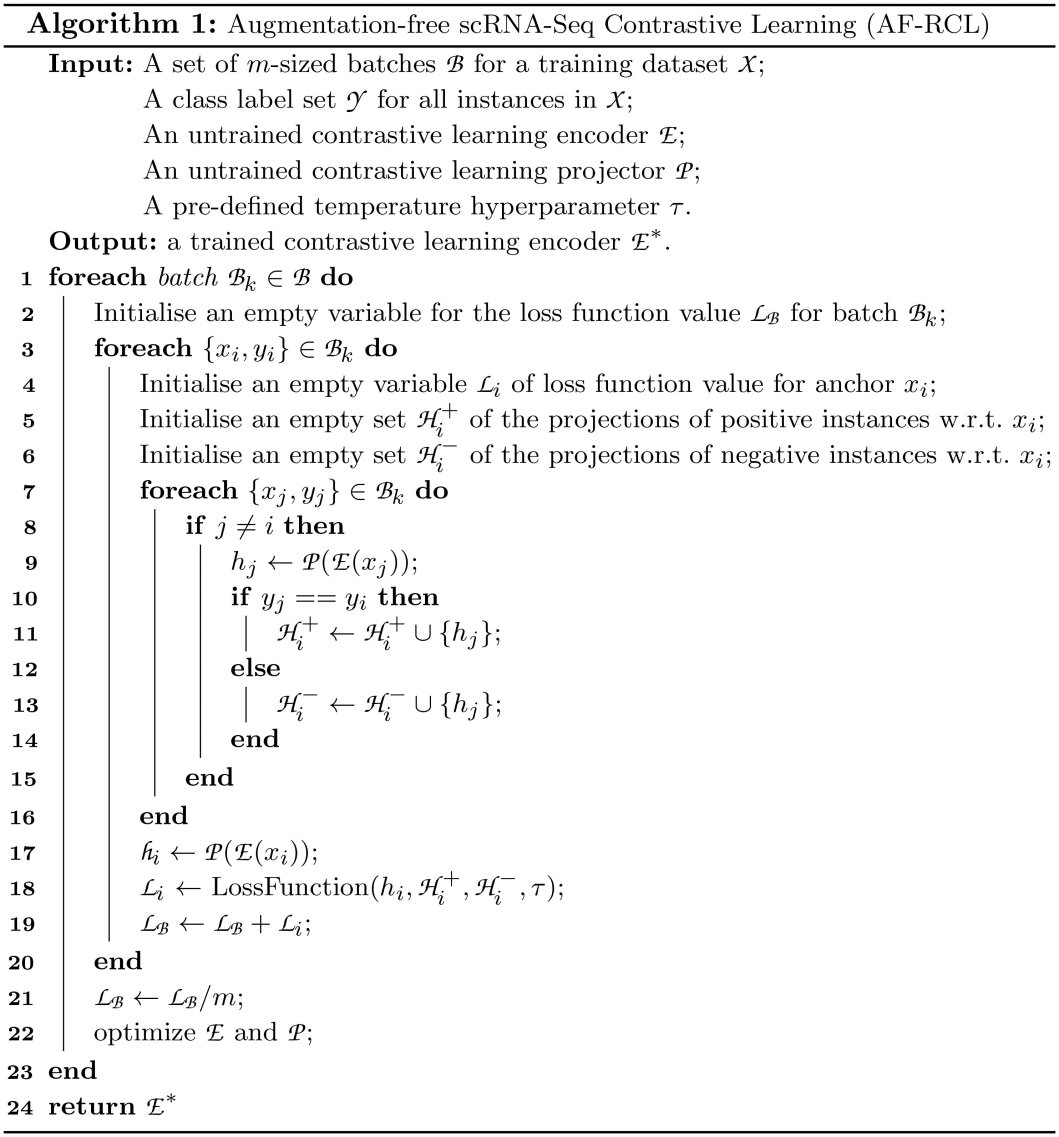


We proposed a new contrastive learning loss function L, which was modified based on the conventional supervised contrastive learning loss function to alleviate overfitting issues—a major factor reducing the predictive performance of contrastive learning-based cell-type identification methods (Alsaggaf *et al.* 2024). As shown in Equation (1), for each target instance $x_{i}$, $L_{i}$ calculates the loss function value by computing the similarities of its projection $h_{i}$ to the projections of all its corresponding positive instances respectively. In terms of each positive instance $x_{q}$, the similarity (i.e. $e^{F(h_{i},h_{q})/\tau }$) of its projection $h_{q}$ to the corresponding target instance’s projection $h_{i}$ is divided by the summation of $e^{F(h_{i},h_{q})/\tau }$ and the summation of similarities of $h_{i}$ with its all corresponding negative instances, where F(·) is the cosine similarity. The denominator of Equation (1) is the major difference to the conventional supervised contrastive loss function [Equation (S1), available as supplementary data at *Bioinformatics* online], which uses the similarities of the target instance projection $h_{i}$ with all positive and negative instances as the denominator, leading to higher overall loss function values.

(1) $$L_{i}=\frac{-1}{\left|H_{i}^{+}\right|}\sum_{h_{q}\in H_{i}^{+}}\;\text{log}\;\frac{e^{F(h_{i},h_{q})/\tau }}{e^{F(h_{i},h_{q})/\tau }+\sum_{h_{l}\in H_{i}^{-}}e^{F(h_{i},h_{l})/\tau }}$$

### 2.2 Computational experiments

We used 18 human and mouse scRNA-Seq datasets selected from Abdelaal *et al.* (2019) and Chen *et al.* (2020a) to evaluate the predictive performance of the proposed AF-RCL method. All those 18 selected scRNA-Seq datasets include sufficient numbers of annotated cells for all individual cell-types, as introduced in Alsaggaf *et al.* (2024). Table 1 shows the details of the 18 scRNA-Seq datasets. For pre-processing, we log-transformed all the 18 original scRNA-Seq expression profiles. For each of the 18 datasets, 20% was treated as a held-out validation set that was used for model selection, the remaining 80% was used to conduct contrastive learning in a 5-fold cross-validation manner. All splits were made on cells and in a stratify manner making sure the distribution of cell-types is approximately preserved in all splits. The batch size was set to 64 (m=64) and batch normalisation was used for training stability. Following recent contrastive learning-based works in scRNA-Seq analysis (Ma and Pellegrini 2020, Ciortan and Defrance 2021, Xu *et al.* 2022, Wan *et al.* 2022), we used relatively small networks comparing to the layer sizes used in Chen *et al.* (2020b) and Khosla *et al.* (2020). We used a four-layer multi-layer perceptron encoder with three 1024-dimensional hidden layers and a 512-dimensional output layer, i.e. representation layer. The projection head consists of a single 256-dimensional hidden layer and a 128-dimensional output layer. ReLU activation was used in both networks. The training was optimized using Adam with a learning rate of $10^{-3}$ and weight decay $10^{-6}$. The number of training epochs was set to 500 and the temperature hyperparameter was set to 0.1 (τ=0.1) according to Khosla *et al.* (2020). After every 5 epochs, we used the frozen encoder to transform the training folds and the held-out validation set into feature representations. An SVM classifier was trained on the transformed training folds to predict the cell-types of the validation set instances. We conducted grid search to select the optimal hyperparameters for Support Vector Machine (SVM). We selected the optimal encoder that its feature representations led to the highest Matthews correlation coefficient (MCC) value. The implementation is conducted using Pytorch (Paszke *et al.* 2019) and Scikit-learn (Pedregosa *et al.* 2011). We used three metrics in multi-class settings, i.e. MCC, F1 score, and accuracy score to evaluate the predictive performance of the cell-type identification methods. MCC evaluates the performance of models with considering the imbalanced distributions of each individual binary classes. As shown in Equation (2), *n* is the number of instances, $p=\sum_{i}^{n}I_{\left({\hat{y}}_{i}=y_{i}\right)}$ is the number of instances correctly predicted, where ${\hat{y}}_{i}$ is the predicted cell-type of the $i^{th}$ cell, and $y_{i}$ is the ground truth. $I_{\left({\hat{y}}_{i}=y_{i}\right)}$ is an indicator function returning the value of 1 if ${\hat{y}}_{i}=y_{i}$ and the value of 0 otherwise. $n_{c}=\sum_{i}^{n}I_{\left(y_{i}=c\right)}$ is the number of times class *c* occurred, and $p_{c}=\sum_{i}^{n}I_{\left({\hat{y}}_{i}=c\right)}$ is the number of times class *c* was predicted. F1 score evaluates the performance of models on predicting instances bearing positive labels. As shown in Equation (3), the subscript *c* indicates the positive class and |C| is the number of classes (i.e. cell-types). Accuracy score [Equation (4)] evaluates the performance of models by considering the percentage of cells whose types are correctly predicted.

(2) $$\text{MCC}=\frac{n\times p-\sum_{c}^{C}n_{c}\times p_{c}}{\sqrt{\left(n^{2}-\sum_{c}^{C}p_{c})\times (n^{2}-\sum_{c}^{C}n_{c}\right)}}$$

 

(3) $${F1}_{\text{macro}}=\frac{1}{\left|C\right|}\sum_{c}^{C}\frac{2\times {\text{TP}}_{c}}{2\times {\text{TP}}_{c}+{\text{FP}}_{c}+{\text{FN}}_{c}}$$

 

(4) $$\text{ACC}=\frac{1}{n}\sum_{i}^{n}I_{\left({\hat{y}}_{i}=y_{i}\right)}$$

**Table 1.** The characteristics of the 18 scRNA-Seq datasets.

Dataset name	Organ/tissue	#Genes	#Cells	#Classes	Ref.
Segerstolpe	Human pancreas	22 757	2133	13	Abdelaal *et al.* (2019)
Muraro	Human pancreas	18 915	2122	9	Abdelaal *et al.* (2019)
PBMCBench 10Xv2	Human PBMC	22 280	6444	9	Abdelaal *et al.* (2019)
PBMCBench Drop-Seq	Human PBMC	19 922	3222	9	Abdelaal *et al.* (2019)
PBMCBench 10Xv3	Human PBMC	21 905	3222	8	Abdelaal *et al.* (2019)
PBMCBench Seq-Well	Human PBMC	21 059	3176	7	Abdelaal *et al.* (2019)
PBMCBench inDrop	Human PBMC	17 159	3222	7	Abdelaal *et al.* (2019)
Xin	Human pancreas	33 889	1449	4	Abdelaal *et al.* (2019)
Baron Mouse	Mouse pancreas	14 861	1886	13	Abdelaal *et al.* (2019)
Quake_Smart-seq2 Lung	Mouse lung	19 390	1676	11	Chen *et al.* (2020a)
Adam	Mouse kidney	23 797	3660	8	Chen *et al.* (2020a)
Romanov	Mouse hypothalamus	21 143	2881	7	Chen *et al.* (2020a)
Quake_10x Limb_Muscle	Mouse limb muscle	16 512	3909	6	Chen *et al.* (2020a)
Quake_Smart-seq2 Limb_Muscle	Mouse limb muscle	18 320	1090	6	Chen *et al.* (2020a)
Quake_Smart-seq2 Diaphragm	Mouse diaphragm	17 973	870	5	Chen *et al.* (2020a)
Klein	Mouse embryonic stem cell	24 047	2717	4	Chen *et al.* (2020a)
Quake_10x Bladder	Mouse bladder	16 867	2500	4	Chen *et al.* (2020a)
Quake_Smart-seq2 Trachea	Mouse trachea	19 992	1350	4	Chen *et al.* (2020a)

## 3 Results

### 3.1 AF-RCL successfully learned a type of discriminative feature representations that improved the predictive performance of original scRNA-Seq expression profiles

**Figure 2.**
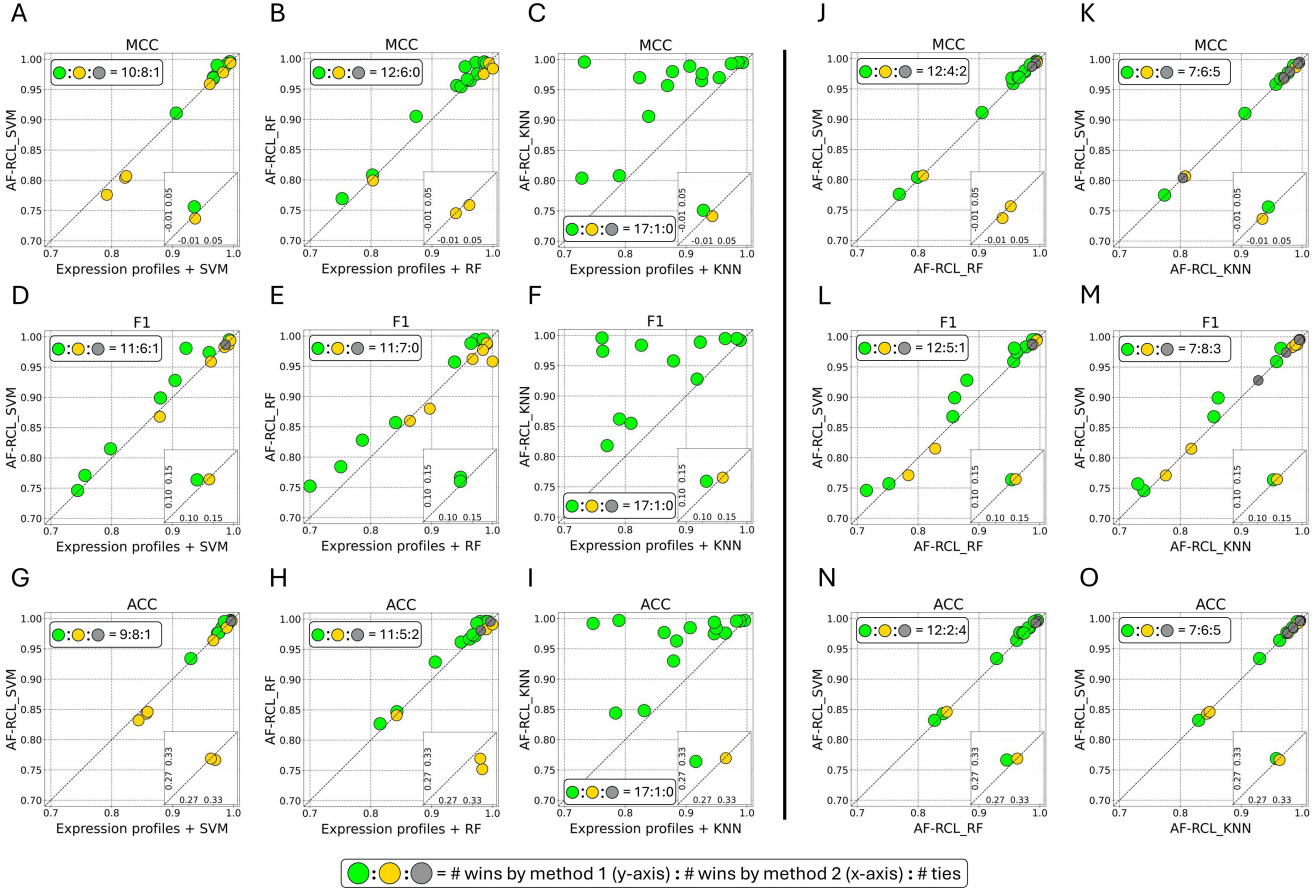
(A–I) The pairwise comparisons between AF-RCL working with three different classifiers and the performance obtained by training those classifiers directly using original scRNA-Seq expression profiles after being log-transformed. (J–O) The pairwise comparisons between AF-RCL working with SVM and other two classifiers. The three numbers in each legend respectively denote the number of wins by the first method on the *y*-axis, the number of wins by the second method on the *x*-axis, and the number of ties.

We first evaluated the predictive performance of the AF-RCL-learned discriminative feature representations using three types of classification algorithms, i.e. support vector machine, random forests, and *k*-nearest neighbours. Figure 2A–I shows pairwise comparisons between the AF-RCL-learned feature representations and the original scRNA-Seq expression profiles, which were log-transformed before being directly used to train the three classifiers, where each figure shows the number of datasets in which the AF-RCL-learned feature representations outperformed the original scRNA-Seq expression profiles and *vice versa*, in addition to the number of ties. Hereafter, all scatterplots follow the same format. In general, the AF-RCL-learned feature representations obtained higher MCC values, F1 scores, and ACC values in the majority of the 18 experimental datasets when using all three types of classifiers. For example, as shown in Fig. 2A, D, and G, AF-RCL_SVM outperformed SVM using original expression profiles in 10, 11, and 9 datasets according to MCC values, F1 scores, and ACC values, respectively. Analogously, as shown in Fig. 2B, E, and H, AF-RCL_RF obtained higher MCC values, F1 scores, and ACC values than RF using original expression profiles in 12, 11, and 11 datasets, respectively. AF-RCL_KNN also outperformed KNN using original expression profiles in almost all datasets (i.e. 17 out of 18) according to all three different metrics, as shown in Fig. 2C, F, and I.

We further compared the predictive performance of AF-RCL_SVM, AF-RCL_RF, and AF-RCL_KNN. In general, AF-RCL_SVM obtained the overall highest predictive accuracy. As shown in Fig. 2J and K, AF-RCL_SVM obtained higher MCC values than AF-RCL_RF and AF-RCL_KNN in 12 and 7 datasets, respectively. AF-RCL_RF and AF-RCL_KNN only outperformed AF-RCL_SVM in 4 and 6 datasets, respectively. In terms of F1 scores, as shown in Fig. 2L and M, AF-RCL_SVM outperformed AF-RCL_RF and AF-RCL_KNN in 12 and 7 datasets, respectively, though AF-RCL_KNN performed better in slightly more datasets (i.e. 8 out of 18 datasets) than AF-RCL_SVM. In terms of ACC values, as shown in Fig. 2N and O, AF-RCL_SVM outperformed AF-RCL_RF in the majority of the experimental datasets (i.e. 12 out of 18), whilst it also outperformed AF-RCL_KNN in more datasets, i.e. 7 out of 18. Therefore, hereafter, we denote the AF-RCL_SVM method as AF-RCL, and we denote SVM using original expression profiles as the benchmark method.

### 3.2 AF-RCL successfully outperformed other state-of-the-art contrastive learning-based cell-type identification methods

We further compared AF-RCL with other state-of-the-art contrastive learning cell-type identification methods, i.e. Sup-GsRCL, Sup-RM5000-RCL, Self-GsRCL, and Self-RM3000-RCL, which were all proposed in Alsaggaf *et al.* (2024). The first two methods respectively use Gaussian noise augmentation and random genes masking strategies to create sample views as inputs for the conventional supervised contrastive learning settings, whilst the last two methods adopt the same two types of data augmentation strategies, respectively, but follow the conventional self-supervised contrastive learning settings.

**Figure 3.**
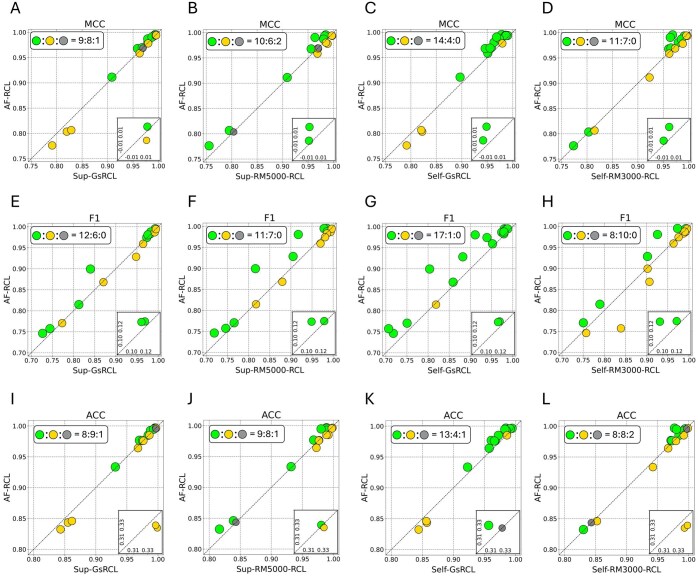
(A-L) The pairwise comparisons between AF-RCL and other contrastive learning-based cell-type identification methods according to MCC values, F1 scores and ACC values.

Figure 3 shows the pairwise comparisons between AF-RCL and each contrastive learning method among those 18 datasets. As shown in Fig. 3A, E, and I, AF-RCL obtained higher MCC values, F1 scores, and ACC values than Sup-GsRCL in 9, 12, and 8 datasets, respectively. Analogously, as shown in Fig. 3B, F, and J, AF-RCL outperformed Sup-RM5000-RCL in 10, 11, and 9 datasets, according to MCC values, F1 scores, and ACC values, respectively. AF-RCL also outperformed Self-GsRCL in the vast majority of the 18 datasets. As shown in Fig. 3C, G, and K, the former obtained higher MCC values, F1 scores, and ACC values in 14, 17, and 13 datasets, respectively. In terms of Self-RM3000-RCL, as shown in Fig. 3D, H, and L, AF-RCL obtained higher MCC values in the majority of the datasets, i.e. 11 out of 18. Although it obtained higher F1 scores in slightly fewer datasets than Self-RM3000-RCL (i.e. 8:10), both methods respectively obtained higher ACC values in 8 out of 18 datasets.

### 3.3 AF-RCL successfully outperformed other machine learning-based cell-type identification methods

We also compared AF-RCL with other recently proposed machine learning-based cell-type identification methods, i.e. scPred (Alquicira-Hernandez *et al.* 2019), ACTINN (Ma and Pellegrini 2020), SingleCellNet (Tan and Cahan 2019), and scGPT (Cui *et al.* 2024). scPred adopts the conventional principal component analysis method to extract informative feature representations, which are then used to train SVM classifiers for identifying different cell-types. ACTINN trains neural networks classifiers directly on pre-processed scRNA-Seq expression profiles to predict different cell-types. SingleCellNet transforms scRNA-Seq expression profiles into a binary matrix derived by pairwise comparisons of selected genes on a per-cell basis, the binary matrix is then used to train random forest classifiers for identifying different cell-types. scGPT is a foundation model for single-cell biology that is based on a generative pre-trained transformer across a repository of over 33 million human cells. It provides fine-tuning pipelines with task-specific objectives, designed to facilitate the application of scGPT across a range of tasks. Since scGPT was trained only using human scRNA-Seq data, we fine-tuned the pre-trained scGPT model for those 8 individual human scRNA-Seq datasets and compared its predictive performance with AF-RCL.

**Figure 4.**
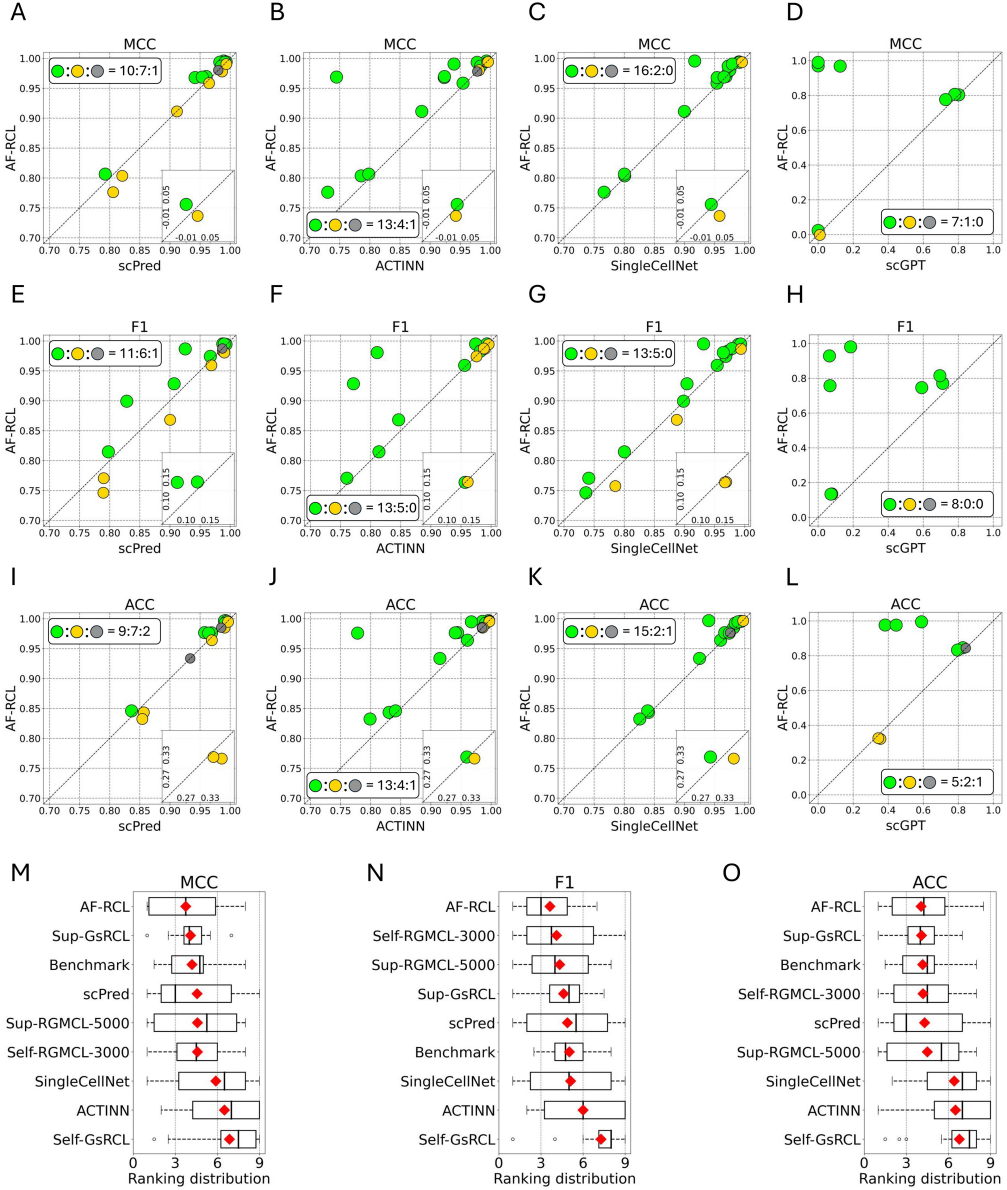
(A–L) The pairwise comparisons between AF-RCL and other machine learning-based cell-type identification methods, where only human scRNA-Seq datasets are used to compare AF-RCL with scGPT. (M–O) The boxplots showing the ranking distributions of nine different methods, where the average rankings are denoted by the red diamond symbol.

In general, AF-RCL outperformed all other cell-type identification methods. As shown in Fig. 4A, E, and I, among those 18 datasets, AF-RCL obtained higher MCC values, F1 scores, and ACC values than scPred in 10, 11, and 9 datasets, respectively. Analogously, AF-RCL obtained higher MCC values, F1 scores, and ACC values than ACTINN all in 13 out of 18 datasets, as shown in Fig. 4B, F, and J. AF-RCL also outperformed SingleCellNet in the majority of those 18 datasets, i.e. 16, 13, and 15 datasets for MCC values, F1 scores, and ACC values, respectively. In addition, AF-RCL successfully outperformed scGPT in almost all human datasets according to MCC values and F1 scores. It also obtained higher ACC values than scGPT in 5 out of 8 human datasets, as shown in Fig. 4D, H, and L.

We further compared the predictive performance of all those cell-type identification methods except scGPT by considering their average rankings obtained over all 18 datasets. The method that showed the best predictive performance is ranked as first, whilst the method that showed the worse predictive performance is ranked as ninth. When more than one method showed the same predictive performance, all those methods obtained the same ranking. Figure 4M–O shows the boxplots of the ranking distributions of all those nine cell-type identification methods according to all three metrics. It is clear that AF-RCL is the overall best method, due to its top rankings according to MCC, F1, and ACC values. Sup-GsRCL is the second best method, as it was ranked in second places according to both MCC and ACC values, though Self-RGMCL-3000 was also ranked in the second place according to F1 scores.

## 4 Discussion

### 4.1 The modified supervised contrastive learning loss function plays a crucial role in the success of AF-RCL

**Figure 5.**
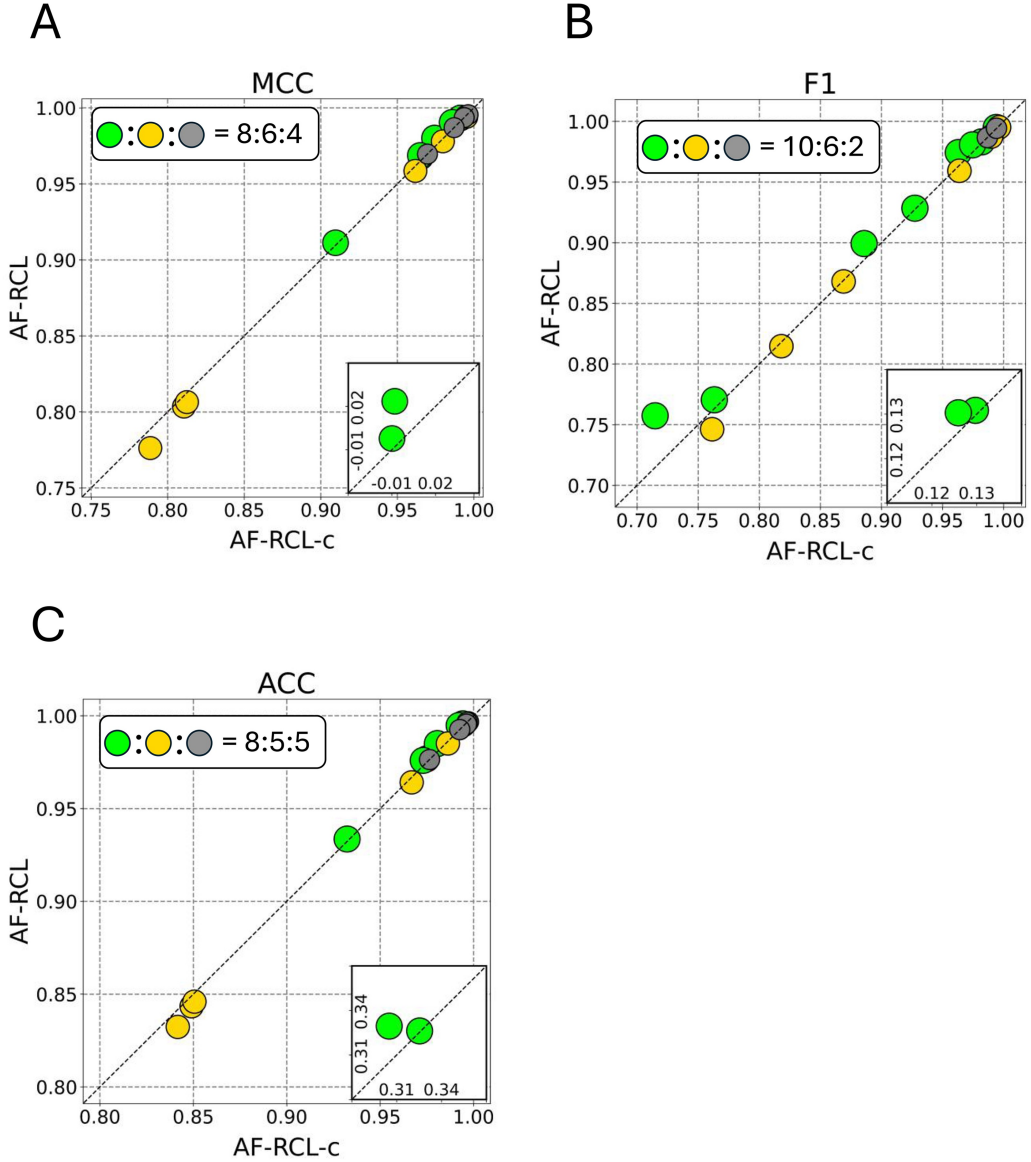
(A-C) The pairwise comparisons between AF-RCL and AF-RCL-c according to MCC values, F1 scores and ACC values.

We investigated the differences between the modified loss function [Equation (1)] and the conventional supervised contrastive learning loss function, as shown in Equation (S1), available as supplementary data at *Bioinformatics* online, where the cosine similarity of a target instance and its corresponding positive instance pairs are normalised by the cosine similarity of the target instance and its both positive and negative instance pairs. We used the conventional supervised contrastive loss function to work with the proposed augmentation-free view creation strategy, denoted as AF-RCL-c. We then conducted head-to-head comparisons between the predictive performance of AF-RCL and AF-RCL-c over all 18 datasets. In general, the modified loss function leads to better predictive performance than the conventional loss function. As shown in Fig. 5A–C, AF-RCL obtained higher MCC values than AF-RCL-c in 8 out of 18 datasets, whilst both methods obtained the same MCC values in 4 datasets. AF-RCL also obtained higher F1 scores and ACC values in 10 and 8 datasets, respectively. AF-RCL-c merely obtained higher MCC values, F1 scores, and ACC values in 6, 6, and 5 datasets, respectively.

### 4.2 AF-RCL learns better contrastive learning-derived feature representations bearing good trade-off between uniformity and tolerance

We further discussed different scRNA-Seq contrastive learning methods by considering the trade-off between uniformity (Wang and Isola 2020) and tolerance (Wang and Liu 2021). In general, a good contrastive learning method should derive optimal feature representations, i.e. locally clustered and globally separated, to improve the performance against downstream tasks. This could be achieved by maintaining two crucial properties, i.e. uniformity and tolerance. The former aims to uniformly distribute the feature representations in a hypersphere, whilst the latter aims to preserve the local semantic information. However, finding a good trade-off between those two properties is challenging (Wang and Liu 2021), since an excessive improvement of uniformity will break the local semantic structure, while only improving tolerance will break the global structure. Therefore, the best contrastive learning method should obtain the highest values in both uniformity and tolerance simultaneously, denoted by the highest product value.

Uniformity is defined as how close the derived feature representations to a uniform distribution in a hypersphere. As shown in Equation (5), it is measured by the logarithm of the average pairwise Gaussian potential, where *t* is a constant and we set t=2 as suggested by Wang and Isola (2020). For convenience, we negated uniformity values, thus larger values mean better results. Tolerance is defined as the local density of semantically related instances given a type of feature representations derived by a contrastive learning method. As shown in Equation (6), it is measured by the average cosine similarity of the transformed instances bearing the same class, where $E^{*}$ is a trained contrastive learning encoder.

(5) $$\text{Uniformity}=-\text{log}\;E_{x_{i},x_{j}∼X}\left[e^{-t\|E^{*}(x_{i})-E^{*}(x_{j}){\|}_{2}^{2}}\right]$$

 

(6) $$\text{Tolerance}=E_{x_{i},x_{j}∼X}\left[F(E^{*}(x_{i}),E^{*}(x_{j}))\cdot I_{\left(y_{i}=y_{j}\right)}\right]$$

**Figure 6.**
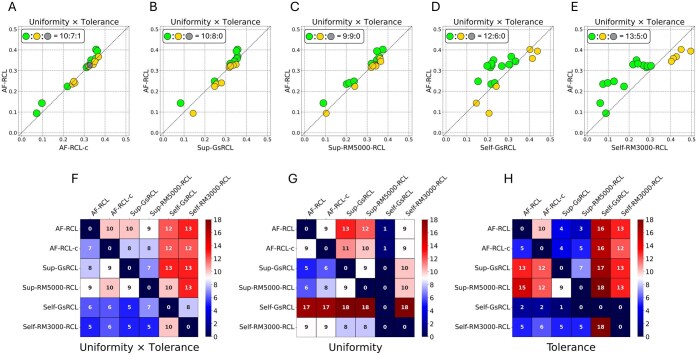
(A–E) The scatterplots showing the pairwise comparisons of the products of uniformity and tolerance values between AF-RCL and other contrastive learning-based cell-type identification methods. (F–H) The heatmaps showing the pairwise comparisons of different contrastive learning-based cell-type identification methods according to the numbers of tasks where each method obtained higher values of the corresponding metrics.

Figure 6A–E shows the pairwise comparisons of the product of uniformity and tolerance values between AF-RCL and other contrastive learning methods. In general, AF-RCL learned better feature representations than AF-RCL-c, Sup-GsRCL, Self-GsRCL, and Self-RM3000-RCL, because the former obtained higher product values in more datasets (i.e. 10, 10, 12, and 13 datasets). Both AF-RCL and Sup-RM5000-RCL obtained the highest product values in the same number of datasets, i.e. 9 out of 18.

Figure 6F, G, and H shows three heatmaps to present the pairwise comparisons between different methods according to uniformity, tolerance and their product values. As shown in Fig. 6F, AF-RCL obtained higher product values in more datasets than all other methods except Sup-RM5000-RCL. In terms of the uniformity metric, as shown in Fig. 6G, Self-GsRCL is the best method as it obtained higher uniformity values than other contrastive learning methods in almost all datasets. However, it failed to learn semantically local distributions, because its tolerance values are lower than all other contrastive learning methods in almost all datasets. AF-RCL is the second-best method for obtaining good uniformity values. It obtained higher uniformity values in more datasets than Sup-GsRCL and Sup-RM5000-RCL, whilst obtaining higher uniformity values in the same number of datasets as AF-RCL-c and Self-RM3000-RCL. In terms of the tolerance metric, as shown in Fig. 6H, Sup-RM5000-RCL is the best method, since it obtained higher tolerance values in more datasets than all other contrastive learning methods. However, it failed to obtain higher uniformity values in more datasets than all other methods except Self-RM3000-RCL. The second-best method for obtaining good tolerance values is Sup-GsRCL, which only failed to obtain higher tolerance values in more datasets than Sup-RM5000-RCL. However, it also obtained poor uniformity values, as shown in Fig. 6G. AF-RCL is the third-best method since it also obtained higher tolerance values in more datasets than AF-RCL-c, Self-GsRCL and Self-RM3000-RCL.

## 5 Conclusion

In this work, we proposed a novel AF-RCL method that not only obtained state-of-the-art predictive accuracy of automatic cell-type identification tasks but also demonstrated its advantages in learning better distributions bearing good uniformity and local semantic distributions. Future research directions would focus on extending the applications of the proposed AF-RCL method to other scRNA-Seq analysis tasks (e.g. data integration and batch effects removal) and developing new self-supervised AF-RCL methods.

## Supplementary Material

btaf437_Supplementary_Data

## Data Availability

The scRNA-Seq datasets used in this work can be downloaded from https://doi.org/10.5281/zenodo.8087611, and the pre-trained AF-RCL encoders can be downloaded from https://doi.org/10.5281/zenodo.15109736.

## Code availability

The source code is available at https://doi.org/10.6084/m9.figshare.28830311.v1 and at https://github.com/ibrahimsaggaf/AFRCL.
