# Supplementary material for: Less is more: improving cell-type identification with augmentation-free single-cell RNA-Seq contrastive learning
Source: Bioinformatics. 2025 Aug 5;41(9):btaf437. doi: 10.1093/bioinformatics/btaf437 (PMC12417077; doi:10.1093/bioinformatics/btaf437)
Supplement: btaf437_Supplementary_Data [file btaf437_supplementary_data.zip › supplementary_file_1.pdf]

Supplementary File 1

# Less is more: Improving cell-type identification with augmentation-free single-cell RNA-Seq contrastive learning

Ibrahim Alsaggaf [1], Daniel Buchan [2], Cen Wan [1,*]

[1] School of Computing and Mathematical Sciences, Birkbeck, University of London, United Kingdom

[2] Department of Computer Science, University College London, United Kingdom

[*] Corresponding author cen.wan@bbk.ac.uk

## 1 The conventional supervised contrastive learning loss function

The conventional supervised contrastive learning loss function [1] was an extension of the self-supervised contrastive learning function [2] to supervised learning settings. As shown in Equation S1, $\mathcal{L}_i$ is the loss value for the projection of the i$^{th}$ instance $h_i$, where $\mathcal{H}_i^+$ is the set of all projected instances that belong to the same class as $h_i$ (i.e. positives). $\mathcal{H}_i$ denotes the set of all projected instances except $h_i$ (i.e. positives and negatives). $\mathcal{F}(\cdot)$ is the cosine similarity and $\tau$ is a temperature hyper-parameter.

$$\mathcal{L}_i = \frac{-1}{|\mathcal{H}_i^+|} \sum_{h_q \in \mathcal{H}_i^+} log \frac{e^{\mathcal{F}(h_i, h_q)/\tau}}{\sum_{h_l \in \mathcal{H}_i} e^{\mathcal{F}(h_i, h_l)/\tau}} \tag{S1}$$

## References

[1] Prannay Khosla et al. Supervised Contrastive Learning. In Proceedings of the 34th International Conference on Neural Information Processing Systems, pages 18661–18673, 2020.

[2] Ting Chen et al. A simple framework for contrastive learning of visual representations. In Proceedings of the 37th International conference on machine learning, pages 1597–1607, 2020.
